# Case report: An infantile lethal form of Albright hereditary osteodystrophy due to a *GNAS* mutation

**DOI:** 10.1002/ccr3.1739

**Published:** 2018-08-16

**Authors:** Valérie Leclercq, Valérie Benoit, Damien Lederer, Melanie Delaunoy, Marcela Ruiz, Claire de Halleux, Olivier Robaux, Catherine Wanty, Isabelle Maystadt

**Affiliations:** ^1^ Centre de Génétique Humaine Institut de Pathologie et de Génétique Gosselies Belgium; ^2^ Centre de Génétique Humaine ULB Hôpital Erasme Bruxelles Belgium; ^3^ Département de Pédiatrie Grand Hôpital de Charleroi Charleroi Belgium

**Keywords:** Albright's hereditary osteodystrophy, GNAS mutation, inactivating PTH/PTHrP signaling disorders, pseudohypoparathyroidism, severe phenotype

## Abstract

Germline loss‐of‐function *GNAS* mutations are associated with multiple phenotypes, depending on the parental origin of the mutant allele. Here, we describe an infantile lethal form of atypical pseudohypoparathyroidism type 1a or 1c with severe Albright's hereditary osteodystrophy phenotype, underlying the extremely variable expressivity of this syndrome.

## Graphical abstract

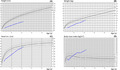

## 1 INTRODUCTION

The *GNAS* gene (MIM#139320) is located at 20q13.32. It encodes for a G alpha subunit protein, which is ubiquitously expressed and plays an important role in many intracellular pathways in response to various stimulations.^1^

Germline inactivating mutations in this gene result in pseudohypoparathyroidism type 1a, 1b, and 1c (PHP‐1a, PHP‐1b, and PHP‐1c), pseudopseudohypoparathyroidism (PPHP), progressive osseous heteroplasia (POH), and osteoma cutis (OC). Albright's hereditary osteodystrophy (AHO) is a phenotypic presentation, which is mainly associated with PHP‐1a and PHP‐1c, and is characterized by a round face, a short stature, subcutaneous ossifications, and brachydactyly type E. Genomic imprinting and tissue‐dependent alternative splicing explain that *GNAS* mutations lead to different phenotypes, mostly depending on whether it affects the paternal or the maternal allele.^2^ The EuroPHP network recently recommended a new classification, which gathers the pseudohypoparathyroidism syndromes under the term inactivating PTH/PTHrP signaling disorders (iPPSD). Accordingly, the GNAS‐related PHP syndromes are proposed to be called iPPSD2.^3^

Thanks to the next‐generation sequencing technologies, more and more inactivating mutations are found in the *GNAS* gene, enlarging the phenotypic spectrum of *GNAS*‐related syndromes. An important interindividual phenotypic variability is described for each clinical entity, even within the same family. However, genotype‐phenotype correlations should be better established, and the role of modifying genetic or environmental factors remains to be demonstrated.

Here, we describe an atypical infantile lethal form of PHP‐1a or PHP‐1c with Albright's hereditary osteodystrophy (AHO) called iPPSD2 following the recently suggested nomenclature, which is the most severe phenotypic presentation reported to date.

## 2 MATERIALS AND METHODS

The patient and his parents were selected for exome sequencing. The analyses were performed on blood samples.

Libraries preparation was performed using Ion Plus Fragment Library Kit (Thermo Fisher Scientific, Waltham, MA). For enrichment of genomic DNA, we used Agilent SureSelectXT Human All Exon V5 (Agilent, Santa Clara, CA).

Targeted regions were then sequenced with Ion Proton (Thermo Fisher Scientific).

More than 80% of ROI (regions of interest) were covered at least 20×. Reads were aligned against the Hg19 reference genome, using Torrent Suite Software (Thermo Fisher Scientific), resulting in a binary alignment map (BAM) file. Variant calling was performed by NextGENe software (SoftGenetics, State College, PA). Analysis was first focused on the *GNAS* gene, which was the first candidate gene related to the clinical presentation. Then, a general study of the entire exome was achieved to exclude any additional pathogenic variant. Sanger sequencing using standard protocols was performed for validation of the identified *GNAS* variant and for parental carriership analysis in blood and saliva.

The MS‐MLPA (methylation‐specific multiplex ligation‐dependent probe amplification) was performed according to the manufacturer's protocol (SALSA^®^MS‐MLPA^®^ kit ME031‐A1 *GNAS*, MCR‐Holland, Amsterdam, the Netherlands).

Written informed consent was obtained from the patient's parents for publication of the medical data and the photographs.

## 3 RESULTS

### 3.1 Clinical report

The proband is the second child of healthy and nonconsanguineous Caucasian parents. His elder brother is healthy.

Pulmonary atresia with ventricular septal defect and severe intrauterine growth retardation were diagnosed by ultrasonography during pregnancy. An amniocentesis was performed, and genetic investigations yielded normal results (standard karyotype, FISH targeting 22q11.2 region, MLPA targeting chromosomic regions associated with velocardiofacial syndrome). There was no toxic exposure during pregnancy.

Emergency cesarean delivery was performed due to fetal distress at 39 weeks 5/7. Birthweight was 2 kg079 (<−4 SD), height was 43.5 cm (−3.5 SD), and head circumference was 33 cm (−1 to −2 SD). Neonatal adaptation was correct.

A congenital hypothyroidism was diagnosed 3 days after birth, and a hormonal complementation was started (first values: TSH 73 mU/L, normal 1.36‐8.8 mU/L and freeT4 7.7 pmol/L, normal 28.3‐68.2 pmol/L).

He had bilateral cryptorchidism, terminal hypospadias, and bilateral inguinal hernias, for which he underwent surgery.

A Blalock‐Taussig (BT) shunt was performed on the right pulmonary artery at the age of 2 months. Due to stenosis of the first shunt, a second BT shunt had to be realized on the left side 3 months later. A short calcium complementation was given twice in an intensive care unit. He had two generalized seizures, not related to hypocalcemia.

The patient also had a posterior glossoptosis and a severe tracheomalacia, which required a tracheostomy at the age of 8 months. Oxygen therapy at home was subsequently initiated. Moreover, the patient suffered from bronchial hyperreactivity, which was treated with inhaled bronchodilators, corticoids, and leukotriene receptor antagonist.

At 10 months, he developed an infectious endocarditis caused by Enterobacter faecalis.

A homograft operation was carried out at 11 months, and the pulmonary arteries had to be dilated 5 months later.

Severe feeding difficulties required a percutaneous gastrostomy and enteral nutrition until the age of 2 years. The patient was also subject to a severe gastroesophageal reflux and chronic diarrhea.

**Figure 1:**
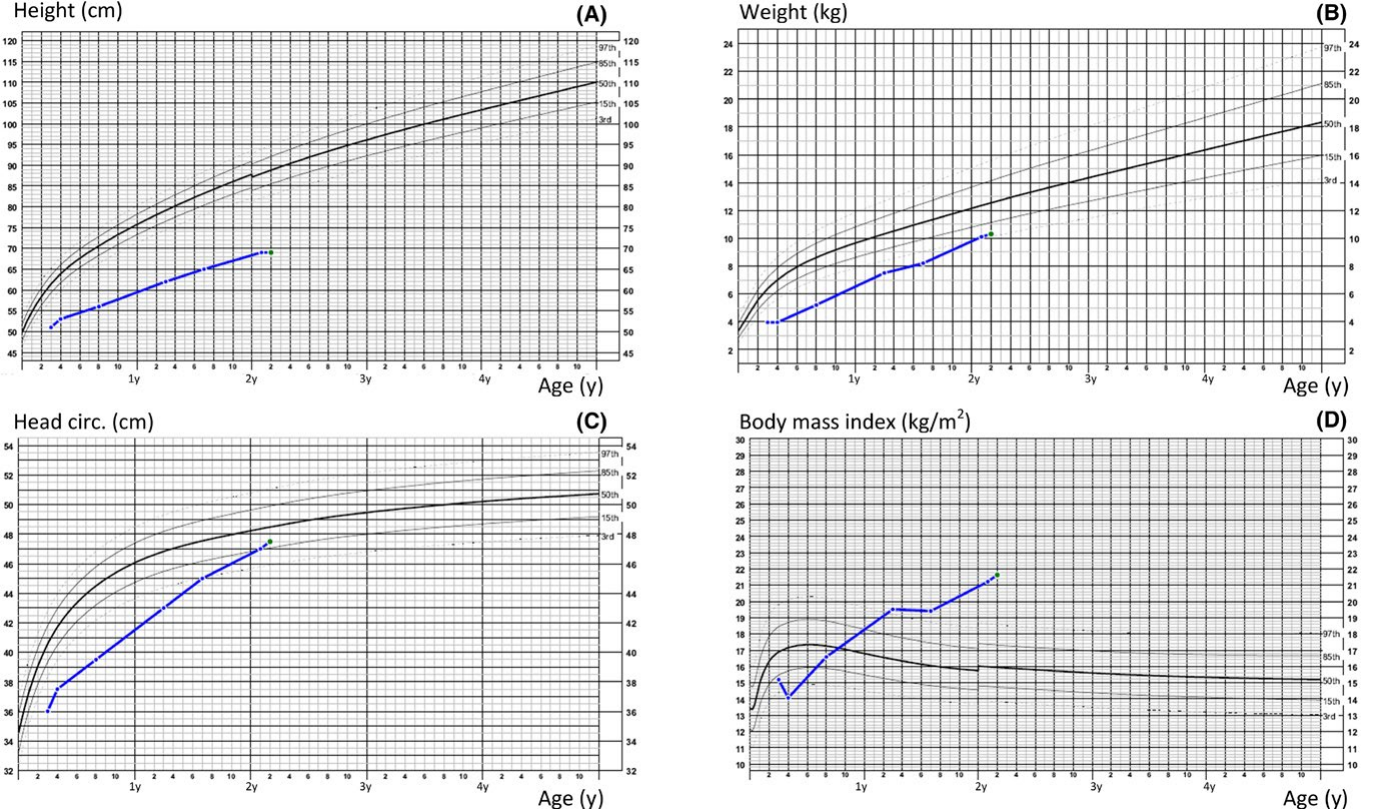
The growth curves of our patient illustrate the severe failure to thrive, which is more pronounced for the height (A) than for the weight (B) and the head circumference (C). The last two parameters tend to normalize with time, contrary to the height. This disharmonic evolution leads to an increasing body mass index (D), which reaches values of morbid obesity after the age of 2 y

The patient had very short stature (−7 SD). The head circumference and weight increased progressively and reached −1.3 SD and −2 SD, respectively. The BMI curve showed a progressive obesity (BMI at 26 months: 21.4, +3.5 SD; Figure 1).

**Figure 2:**
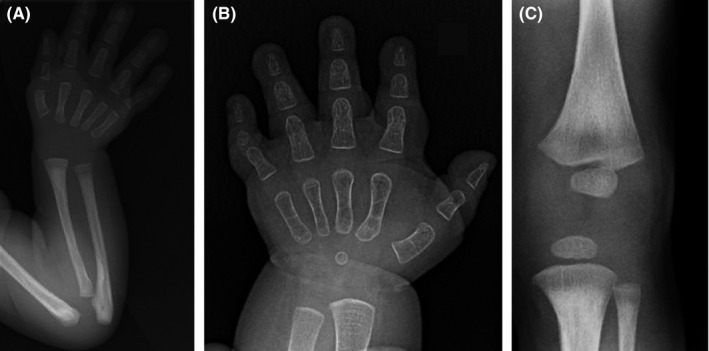
Proband's hand X‐rays at 1.5 mo (A) and 22 mo (B), showing brachymetacarpy and brachydactyly, which are more obvious with age. At 22 mo, the bone age was estimated to be delayed by 1 y. Proband's knee X‐rays at 1.5 mo (C), showing no distinctive sign of acroscyphodysplasia

A skeletal survey carried out at the age of 2 years and 3 months revealed an important osseous rarefaction, delayed bone age, brachymetacarpy, and brachydactyly, but no subcutaneous calcifications (Figure 2).

The patient showed no significant developmental delay and interaction with his relatives was good, although speech capacities were limited due to the tracheotomy.

**Figure 3:**
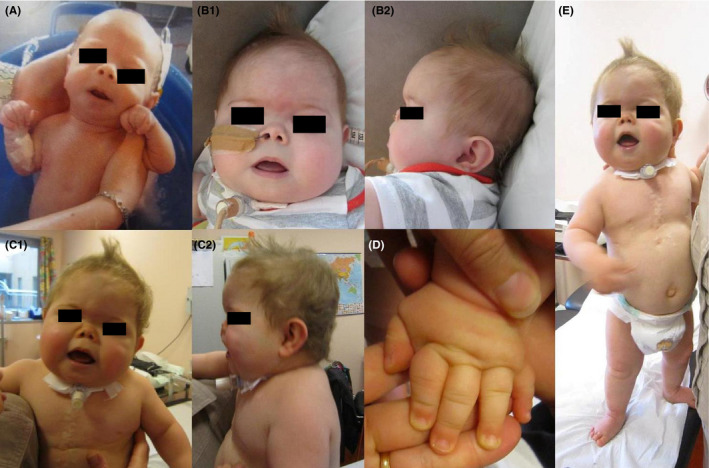
Proband's clinical phenotype: (A) At birth, he had a short stature and moderate facial dysmorphism (short nose with anteverted nostrils, mandibular hypoplasia, and full cheeks). At 16 mo (B1‐2) and at 27 mo (C1‐2, D, E), he showed severe growth retardation, obesity, facial dysmorphism (round face, extremely short nose, prominent tongue, full cheeks), short neck, brachydactyly, and edema of the hands and feet

At the age of 27 months, the clinical examination revealed, besides a significant failure to thrive, a facial dysmorphism (round face, flat nasal bridge, very short nose with anteverted nostrils, narrow choana, full cheeks, prominent tongue, and mandibular hypoplasia). The neck was short and thick. The hands and feet showed an edema as well as brachydactyly, bilateral single transverse palmar crease, and small joints hyperlaxity (Figure 3).

Repeated biological controls revealed lower normal range or slightly decreased serum calcium levels and normal phosphate values. GH and IGF1 dosages were within the normal range at 6 and 26 months. PTH was only once measured at 26 months and showed normal values. We could not find any 25‐OH‐vitamin D dosage (Table 1).

**Table 1:** Phosphocalcic and hormonal dosages in proband's blood samples

	Normal values	6 m	10 m	11 m	18 m	26 m
Ca (mmol/L)	2.18‐2.68		2.15	2.35	1.86	2.18
P (mmol/L)	1.36‐1.74		1.38	1.42	1.52	1.97
PTH (ng/L)	14.9‐56.9					52
GH (µg/L)	0‐0.27	0.18				0.24
IGF1 (ng/mL)	28‐131	101				89

The evolution was marked by a progressive and severe pulmonary arterial hypertension. Finally, the patient suffered from two cardiac arrests in the context of acute pulmonary infection and died at the age of 32 months.

### 3.2 Genetic findings

Standard karyotype and DNA array‐oligo ISCA 180K revealed no chromosomal rearrangements.

**Figure 4:**
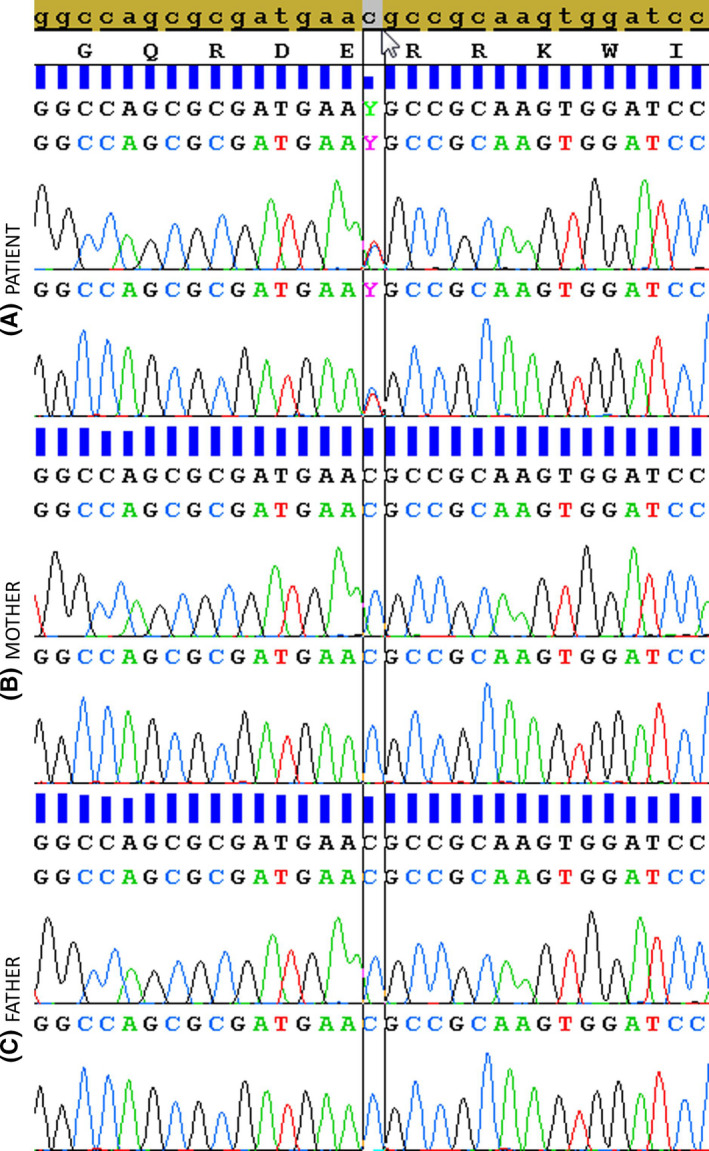
The c.691C>T, p.Arg231Cys mutation in the GNAS gene (NM_000516.4) was detected in the patient's lymphocytes (A), but was absent in the maternal (B) and paternal (C) blood samples

**Figure 5:**
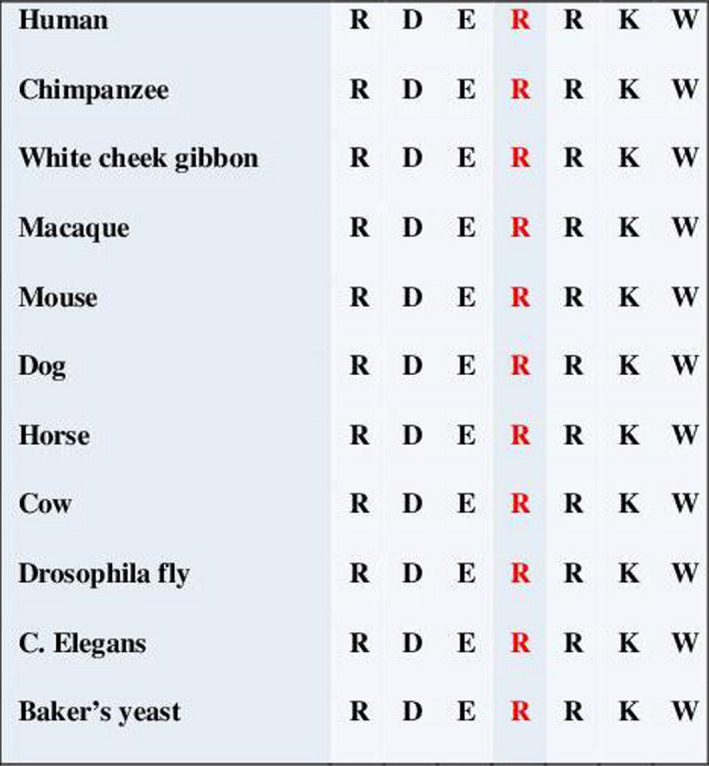
The arginine concerned by the c.691C>T, p.Arg231Cys mutation is highly conserved through the species in the amino acid sequence of the G alpha subunit (reference of the human protein sequence: NP_000507.1)

A trio whole‐exome sequencing showed a heterozygote missense variant in exon 9 of the *GNAS* gene (c.691C>T, p.Arg231Cys [NM_000516.4, NP_000507.1]) in the proband's sample but not in his parents (Figure 4). The de novo variant was confirmed by Sanger sequencing. The variant does not have an entry in the ExAC database, although this locus has a good coverage in gnomAD exomes samples. It concerns a very conserved amino acid (from Saccharomyces Cerevisiae and Caenorhabditis Elegans), and the physicochemical properties of arginine and cysteine are very different (Figure 5). The variant is located in the Guanine nucleotide‐binding protein domain, more precisely in the switch 2 region that is thought to interact with the βγ subunit of the G protein. The prediction programs (SIFT, mutation taster) assign respectively a deleterious and disease causing significance to this p.Arg231Cys variant, which was already reported as pathogenic in the literature.^4^, ^5^

We could not conclude whether the mutation was located on the paternal or maternal inherited allele.

By MS‐MLPA, we excluded an additional pathogenic mechanism within the *GNAS* locus, such as a deletion or an aberrant methylation profile. We could not perform additional studies to evaluate the expression and the activity of the Gsα protein.

To exclude a parental somatic mosaicism for the *GNAS* mutation, a buccal swab was realized in both parents. The *GNAS* c.691C>T variant could not be found in saliva. However, a germinal mosaicism could not be excluded.

## 4 DISCUSSION

The *GNAS* gene encodes for the G alpha subunit (Gsα) of a protein complex called guanine nucleotide‐binding protein (G protein). The G protein is ubiquitously expressed and takes part in a network of intracellular signaling pathways in response to various extracellular signals. This enzyme is mainly involved in the regulation of various hormones production and bone development.

The *GNAS* locus has a highly complex imprinted expression pattern. It encodes for maternally, paternally, and biallelically expressed transcripts that are derived from four alternative first exons. A tissue‐dependent alternative splicing generates two long and two short isoforms of the protein, as well as other noncoding transcripts.

Gsα transcripts are biallelically expressed, except in some tissues such as the renal proximal tubules, thyroid, gonads, and anterior pituitary gland, in which the maternal allele is preferentially expressed. Heterozygous germline inactivating *GNAS* mutations on the maternally transmitted allele lead to type Ia or type Ic pseudohypoparathyroidism (PHP‐1a, PHP‐1c), which is associated with hormonal resistance to parathyroid hormone (PTH), thyroid‐stimulating hormone (TSH), gonadotropins (LH, FSH), growth hormone‐releasing hormone (GHRH), calcitonin, and some neurotransmitters of the central nervous system. Distinction between PHP‐1a and PHP‐1c is based on functional activity of the Gsα in some biochemical assays. Classical manifestations of PHP‐1a and PHP‐1c are primary hypothyroidism without goiter, hypocalcemia with elevated PTH level, short stature due to GH deficiency, and hypogonadism. Mild‐to‐moderate intellectual disability and early‐onset obesity might reflect the Gsα deficiency in imprinted cerebral areas. Albright's hereditary osteodystrophy (AHO) is the phenotype observed in PHP‐1a and PHP‐1c and is characterized by a short stature, a round face, a short neck, limb anomalies (especially brachydactyly of the 4th and 5th metacarpals), and inconstant subcutaneous calcifications. Osteoporosis, osteomalacia, acetabular dysplasia, small epiphysis, and advanced bone age are frequent. Stridor, carpal tunnel syndrome, and sudden loss of vision (due to an enlargement of the sphenoid mucocele) have been reported.

In contrast, when mutation is paternally inherited, it causes pseudopseudohypoparathyroidism (PPHP, with features of AHO but without hormonal resistance) or heterotopic ossifications (progressive osseous heteroplasia or osteoma cutis, without hormonal resistance or AHO manifestations).^6^

It is also demonstrated that obesity (which is progressive during the first years of life) is predominant and more severe in case of maternal transmission, while paternal transmission causes more frequent and severe intrauterine growth retardation.^7^, ^8^

In some cases of pseudohypoparathyroidism (PHP‐1b), the disease is not caused by a mutation in the coding sequence of the maternally inherited *GNAS* gene but by a deletion within the regulatory regions of the *GNAS* locus leading to an imprinting defect.^9^

The various *GNAS*‐related phenotypes are thus mainly explained by imprinting mechanisms.

However, only few genotype‐phenotype correlations were described to explain the very large interindividual phenotypic variability within each *GNAS*‐related clinical entity.^10^ Thiele et al^11^ suggested that missense mutations could lead to a more severe brachymetacarpy and reduced subcutaneous calcifications. Elli et al^12^ hypothesized that mutations in exon 1 could be associated with more ectopic ossifications. Besides imprinting mechanisms, the phenotype may be influenced by environmental or genetic modifying factors, which remain to be identified. Additional mosaic defects, either in *GNAS* or in other genes/chromosomes, could also contribute to the wide phenotypic variability that is observed among patients with *GNAS* mutation.

For our patient, the diagnosis of PHP‐1a or PHP‐1c was hypothesized, based on the association of AHO features (Figures 2 and 3), early‐onset and progressive obesity (Figure 1), and congenital hypothyroidism. Pre‐ and postnatal growth failure might reflect GH deficiency, but IGF1 and GH dosages were normal at the age of 6 and 26 months. A GH stimulation test was planned but could not be performed due to intercurrent medical complications. The distinction between PHP‐1a and PHP‐1c could not specifically be established, as additional studies to evaluate the expression and the activity of the Gsα could not be performed before his premature death. As we could not clearly confirm alteration of calcium metabolism, the diagnostic hypothesis of PPHP could also be discussed.

The PHP/AHO diagnosis was confirmed by the detection of the de novo heterozygous c.691C>T, p.Arg231Cys variant in the *GNAS* gene (NM_000516.4; NP_000507.1). This concerns a very conserved amino acid located in the guanine nucleotide‐binding protein domain, and it has already been reported in the literature. Ahrens et al^4^ identified this variant in a young patient with PPHP and AHO, and demonstrated a reduction in the Gsα activity (58%) by in vitro analysis. The parental origin of the mutation was not identified. Mitsui et al reported the same variant, maternally inherited, in a 7‐year‐old Korean female patient with PHP and acroscyphodysplasia, a distinctive form of metaphyseal dysplasia characterized by metaphyseal deformity and cup‐shaped distal femoral and proximal tibial epiphyses. The patient presented with very short nose, short stature, stubby digits, severe brachydactyly, mild and asymmetric scypho‐deformity of the knees, intellectual impairment, PTH resistance with low‐calcium level but intact PTH, and TSH resistance detected in the neonatal period. She had imperforate anus, intestinal neuronal dysplasia, and syndactyly of right III‐IV and left III‐IV‐V fingers.^5^ The loss‐of‐function c.692G>A, p.Arg231His (NM_000516.4; NP_000507.1) variant, involving the adjacent nucleotide but the same amino acid, was also already described as pathogenic in seven patients from three unrelated families.^13^, ^14^ The five affected children had a classical PHP‐Ia phenotype. The two carrier mothers had attenuated phenotype, possibly due to a paternal origin of the mutated allele, but also attributed to somatic mosaicism for one of them.^15^ Functional studies showed that the p.Arg231His mutation markedly impairs ability of the α subunit to stimulate cAMP accumulation in response to α2‐receptor stimulation.^13^, ^16^ Additional immunofluorescence analyses revealed that the mutation impairs the agonist‐induced translocation of the α subunit from plasma membrane to cytosol.^16^ Finally, transfection assays showed that the mutation impairs tight binding of GTP to Gα, which leads to an activation defect intensified by the presence of activating stimuli.^16^

In our case, we could not determine whether the mutation was located on the paternal or maternal inherited allele. Severe IUGR could be in favor of a paternally inherited variant. However, due to the association of TSH resistance, low‐calcium levels, and early‐onset obesity, it is likely that the mutation is located on the maternal allele, as it was demonstrated for patients with p.Arg231Cys or p.Arg231His mutations when parental samples were available.

According to the new classification proposed by the EuroPHP network, pseudohypoparathyroidism syndromes could now be designated as inactivating PTH/PTHrP signaling disorders (iPPSD). Our patient fulfills the diagnostic criteria, as he shows one major criterion (brachydactyly type E) and 4 minor criteria (TSH resistance, intrauterine and postnatal growth retardation, obesity/overweight, flat nasal bridge, and/or maxillar hypoplasia, and/or round face). Due to the identification of a loss‐of‐function mutation in Gsα, the term iPPSD2 is appropriate.^3^ The new proposed classification simplify the clinical discussion, as it gathers the phenotypic categories of GNAS‐related PHP syndromes (including PHP‐1a, PHP‐1c and PPHP) under the unique iPPSD2 entity.^3^

Although the PHP/AHO (iPPSD2) diagnosis could be established, the phenotype of our patient remains atypical. Indeed, he did not show high‐PTH levels or severe hypocalcemia. However, it is described that the increase in PTH and the decline in calcium levels can be progressive over time, secondary to gradual loss of the expression of paternal Gsα in the maternally imprinted tissues.^2^, ^17^, ^18^ Interestingly, both p.Arg231Cys patients reported in the literature also had intact PTH. Our patient presented with congenital malformations, such as pulmonary atresia with ventricular septal defect, bilateral cryptorchidism, terminal hypospadias, bilateral inguinal hernia, posterior glossoptosis, and severe tracheomalacia, which are not classically described in PHP/AHO. However, the p.Arg231Cys Korean female patient also presented with congenital malformations. Finally, the severe outcome, with a premature death at 32 months, is unusual. Severe presentations of *GNAS*‐related syndromes were already reported. The only child reported with an infantile lethal AHO had a de novo mutation in exon 13 of the *GNAS* gene. He had craniosynostosis, congenital hypothyroidism, hypotonia, AHO's skeletal features, transmission deafness, unilateral choanal atresia, and hydrocephaly. He was originally normocalcemic but after the craniosynostosis surgery, he developed severe hypocalcemia, causing a circulatory shock and a disseminated intravascular coagulation that led to a lethal cerebral infarction.^19^ A second reported child did not have a lethal outcome but a severe form of pseudohypoparathyroidism that is associated with morbid obesity.^20^ He showed TSH and PTH resistance, diagnosed at the age of 3.6 years, and prothrombotic status. A Gsα defect was demonstrated in platelets. The severity of the phenotype was attributed to a compound heterozygous status for two mutations in the *GNAS* gene. Another case of severe pseudohypoparathyroidism type 1b was reported in a child with a broad epigenetic defect without deletion in the *GNAS* region. He presented with neonatal hypothyroidism, high PTH levels, normocalcemia, overweight but normal statural growth, gastroesophageal reflux, asthma, laryngomalacia, auricular septal defect, and macroglossia.^21^ Finally, a few cases of cardiac failure and convulsions likely due to hypocalcemia were reported; however, molecular diagnosis was rarely established.^22^, ^23^

Due to the severity of our case, questions arise as to the possible interaction of modifying genetic or environmental factors, which could worsen the clinical picture.^24^ Through MS‐MLPA, we excluded a deletion in the *GNAS* promotor regions or an abnormal methylation pattern. Using CGH arrays and whole‐exome sequencing, we also excluded the presence of another clear pathogenic copy‐number variation (CNV) or gene variant, which could explain the severe phenotype. In particular, we excluded a *HDAC4* deletion (2q37.3 deletion syndrome), a *STX16* deletion (PHP1b)*,* a mutation in *PRKAR1A, PDE3A,* or *PDE4D* (acrodysostosis), a mutation in *PTH1R* (Blomstrand or Eiken dysplasia), or a mutation in other syndromic causal genes or in genes coding for other intracellular signal transmission effectors. So we failed to identify modifying genetic factors, which could explain the severe phenotype of our patient.

In conclusion, we report on a boy who presented with severe clinical signs of Albright's hereditary osteodystrophy (AHO), congenital hypothyroidism, and severe congenital malformations. PHP/AHO (iPPSD2) diagnosis was established by the identification of a de novo missense mutation (c.691C>T, p.Arg231Cys; NM_000516.4, NP_000507.1) in exon 9 of the *GNAS* gene. The patient died at 32 months from severe pulmonary infection and cardiac arrest. This report illustrates the important phenotypic variability in *GNAS*‐related syndromes and describes the most severe phenotype reported to date. With recent genetic techniques, particularly with genome sequencing, more and more *GNAS* mutations should be found in atypical clinical presentations, extending the phenotypical spectrum of *GNAS*‐related syndromes and maybe improving the genotype‐phenotype correlations.

## CONFLICT OF INTEREST

The authors have declared no conflict of interests.

## AUTHORSHIP

DL, MR, CD, OR, CW, and IM: were involved in patient management. VB, MD, and IM: provided genetic analysis of samples. VL, VB, and IM: wrote the manuscript. OR, CD, DL, and MD: reviewed the manuscript.
